# Carcinome neuroendocrine du sein: à propos d'un cas et revue de la literature

**DOI:** 10.11604/pamj.2016.24.78.8546

**Published:** 2016-05-24

**Authors:** Mariam Affane, Leila Elmorjani, Abdelhamid El Omrani, Fayçal Abbadi, Hanane Rais, Mouna Khouchani

**Affiliations:** 1 Service d'Oncologie-Radiothérapie, CHU Mohammed VI de Marrakech, Maroc; 2 Service d'Anatomo-Pathologie, CHU Mohammed VI de Marrakech, Maroc

**Keywords:** Carcinome neuroendocrine, tumeur du sein, anatomopathologie, traitement, pronostic, Neuroendocrine carcinoma, breast tumor, treatment, prognosis

## Abstract

Les carcinomes neuroendocrines primitifs (CNEP) du sein sont des tumeurs rares. Ils sont actuellement inclus dans la dernière classification de l'OMS des tumeurs du sein. Nous rapportons un cas de localisation mammaire chez une patiente de 39 ans. Il s'agissait d'une tumeur localement avancée ayant nécessité une mastectomie et un curage ganglionnaire axillaire homolatérale. Une chimiothérapie adjuvante était indiquée. L’évolution était marquée par une poursuite évolutive locale. La patiente est décédée dans un tableau de pancytopénie fébrile après une survie d'un an.

## Abstract (en)

Primitive neuroendocrine carcinomas of the breast are rare tumors. They are now included in the latest WHO classification of tumors of the breast. We report the case of a 39-year old patient with tumor located in the breast. It was a locally advanced tumor that required mastectomy and ipsilateral axillary node dissection. Adjuvant chemotherapy was indicated. The evolution was marked by local progression. The patient died in a state of febrile pancytopenia after a one-year survival.

## Introduction

Les tumeurs neuroendocrines primitives du sein sont une forme histologique rare représentant moins de 0,1% de l'ensemble des cancers du sein [1, 2]. Ces tumeurs étaient initialement décrites par Cubilla et al. en 1977; depuis d'autres cas ont été rapportés. Les CNEP mammaires sont actuellement inclus dans la dernière classification de l'OMS des tumeurs du sein [3]. Notre objectif était de discuter à travers un nouveau cas, pris en charge au service d'oncologie-radiothérapie de Marrakech, les particularités anatomo-cliniques, les aspects thérapeutiques et évolutifs de ce cancer.

## Patient et observation

**Figure 1:**
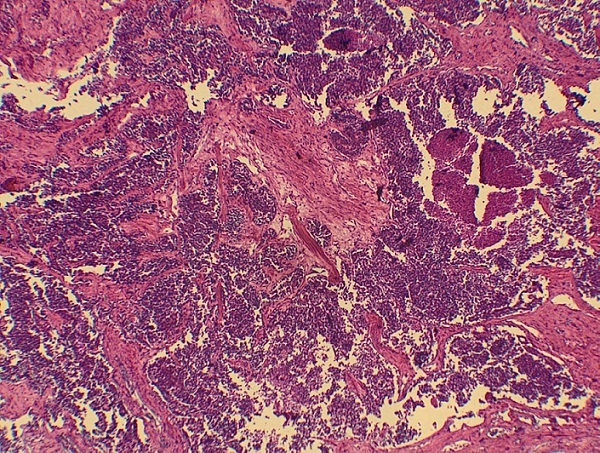
Aspect morphologique (HE) montrant une prolifération carcinomateuse infiltrante d'allure neuroendocrine (x4)

**Figure 2:**
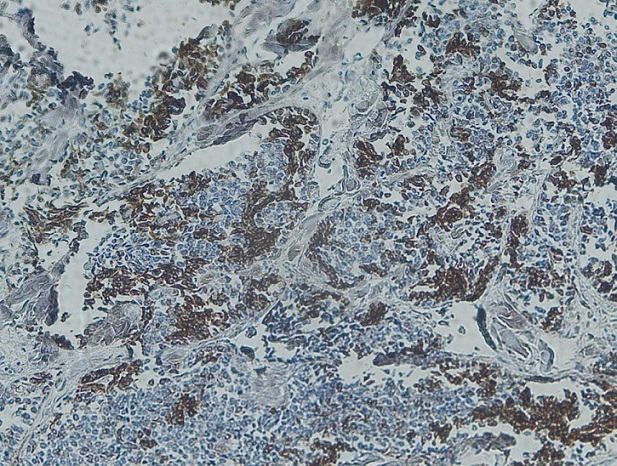
Aspect immunohistochimique montrant une expression à la synaptophysine (x4)

**Figure 3:**
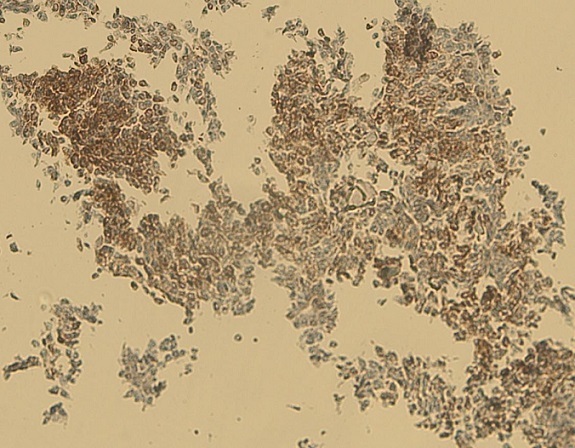
Aspect immunohistochimique montrant une expression à la chromogranine (x4)

**Figure 4:**
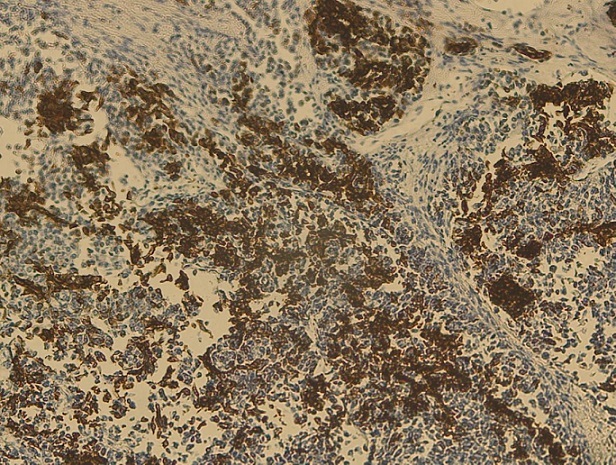
Aspect immunohistochimique montrant une expression à l'anticorps anti-cytokératine

**Figure 5:**
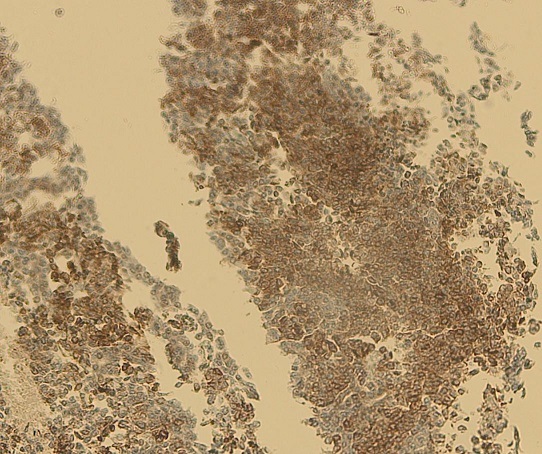
Aspect immunohistochimique montrant une expression au marqueur NSE (x4)

Notre cas concerne une patiente âgée de 39 ans, sans antécédents pathologiques particuliers. Elle a présenté depuis sept mois d'une masse au niveau du sein droit augmentant progressivement de volume avec une adénopathie axillaire homolatérale. L'examen physique retrouvait une masse tumorale de 9cm de grand axe, occupant presque la totalité du sein droit, sans signes inflammatoires en regard et une adénopathie axillaire homolatérale mobile. Le reste de l'examen physique était normal La mammographie et l’échographie mammaires ont montré un placard dystrophique bilatéral avec une formation mammaire droite d'environ 103*104 mm. Une biopsie du nodule droit a été réalisée objectivant une mastopathie proliférante fibro-kystique avec des foyers de discrètes atypies. Une chirurgie de type mastectomie avec un curage axillaire gauche ont été réalisés. L’étude macroscopique a montré que toute la masse mammaire est remplacée par une lésion tumorale diffuse, de consistance ferme faisant 15*12*8cm. A l'examen microscopique, il s'agissait d'un processus néoplasique malin indifférencié et infiltrant évoquant un carcinome avec différenciation endocrine (Figure 1) sans métastase ganglionnaire (0N/9N). Le plan cutané était infiltré. Les berges d'exérèse chirurgicales étaient saines. À l’étude immunohistochimique: la synaptophysine (Figure 2), la chromogranine (Figure 3), la cytokératine (Figure 4) et le marqueur NSE (Figure 5) étaient exprimés. Les récepteurs hormonaux étaient faiblement exprimés (récepteurs à l'estrogène: 10%, récepteurs à la progestérone 15%). L'anticorps anti-Ki67 était également faiblement exprimé (2%). L'expression membranaire des cellules tumorales invasives à l'anticorps anti-HER2 était absente. Le diagnostic retenu alors était celui de carcinome neuroendocrine à grandes cellules. Dans le but d’éliminer une origine secondaire, une échographie abdominale, une radiographie du thorax, une scintigraphie osseuse et un octréoscan ont été demandés. Le bilan s'est avéré normal. Sur la base de ces données, nous avons retenu le diagnostic de carcinome neuroendocrine à grandes cellules primitif du sein. La décision thérapeutique était de faire six cures de chimiothérapie adjuvante à base d’étoposide et cisplatine, puis une une radiothérapie externe et une hormonothérapie adjuvante de type anti-aromatases. L’évolution était marquée par une poursuite évolutive locale après 2 cures. La patiente a bénéficié de huit cures avec une mauvaise réponse clinique. Les complications étaient marquées par une neutropénie de grade 4 et une insuffisance rénale. La patiente est décédée dans un tableau de pancytopénie fébrile après une survie d'un an.

## Discussion

Les carcinomes neuroendocrines mammaires sont rares, représentant moins de 0.1% de tous les cancers mammaires et moins de 1% des tumeurs neuroendocrines. [1]. Ils sont bien définis dans la classification de l'OMS 2003 par leur aspect morphologique similaire aux tumeurs neuroendocrines d'autres sites et par l'immuno-expression de marqueurs neuroendocrines dans plus de 50% du volume tumoral [3]. On décrit quatre groupes: les carcinomes neuroendocrines solides, les carcinoïdes atypiques, les carcinomes à petites cellules et les carcinomes neuroendocrines à grandes cellules [3]. Cette classification exclue les carcinomes mammaires avec différenciation neuroendocrine focale révélée par l'expression d'un marqueur neuroendocrine par des cellules éparses [4, 5]. Dans notre observation, il s'agit d'un carcinome neuroendocrine à grandes cellules. Sur le plan épidémiologique, les carcinomes neuroendocrines surviennent habituellement chez la femme âgée autour de la septième décennie [6]. L'homme peut également être touché [7, 8]. L'aspect clinico-mammographique semble similaire à l'adénocarcinome [9]. Macroscopiquement, les CNEP du sein se présentent sous forme d'une tumeur ronde ou polylobée de couleur jaune blanchâtre, de consistance ferme, ou rarement gélatineuse en cas de composante mucineuse associée [3]. Sur le plan morphologique, les carcinomes neuroendocrines à grands cellules représentent une frontière entre le carcinome neuroendocrine carcinoïde atypique et le carcinome neuroendocrine à petites cellules [10]. Il s'agit d'une morphologie neuroendocrine avec un haut pouvoir mitotique et nécrotique. Les cellules sont grandes au cytoplasme modéré à abondant. Sur le plan immunohistochimique, les carcinomes neuroendocrines sont définis par la présence de plus de 50% de cellules exprimant les marqueurs neuroendocrines. Les cellules neuroendocrines synthétisent en effet des neuropeptides communes (sérotonine, calcitonine) et autres neuropeptides spécifiques «Neurone Specific Enolase (NSE), chromogranine A, synaptophysine »; marqueurs utiles pour démontrer la nature neuroendocrine de la tumeur [11]. Dans notre observation, les 3 marqueurs étaient exprimés. Les récepteurs hormonaux sont rarement présents dans les carcinomes neuroendocrines du sein. Néanmoins, l'expression des récepteurs d’œstrogène et de progestérone dans les carcinomes neuroendocrines d'autres sites a été rapportée. Leur expression dans le sein n'est pas donc la preuve bien déterminée de l'origine mammaire. Ceci dit, La certitude de l'origine mammaire de ces tumeurs repose surtout sur la mise en évidence d'un contingent in situ et sur l'exclusion d'un site extra-mammaire [9].

Dans cette observation, l'origine mammaire a été retenue après avoir éliminé une métastase tumorale d'autres sites par un bilan d'extension. Sur le plan thérapeutique, il n'existe pas de standard thérapeutique. Le traitement est essentiellement chirurgical [12]. Les indications de la chimiothérapie et de la radiothérapie sont les mêmes que pour les autres cancers du sein. Vu la rareté de cette entité, le protocole de chimiothérapie n'est pas standardisé: les carcinomes neuroendocrines du sein sont traités par les uns comme un adénocarcinome du sein et par d'autres comme un carcinome neuroendocrine du poumon [9]. Les carcinomes neuroendocrines à variante solide et les carcinoïdes atypiques sont de meilleur pronostic que les carcinomes neuroendocrines à petites et à grandes cellules. La présence d'un contingent mucineux associé serait un facteur de bon pronostic [13]. Dans notre observation, il existe des facteurs histologiques de mauvais pronostic tels que le caractère indifférencié de la tumeur et l'immuno-expression faible des récepteurs hormonaux.

## Conclusion

Les CNEP du sein sont des tumeurs rares. L’étude de séries plus larges permettra de mieux connaître le profil évolutif et d'adapter le bilan d'extension et la stratégie thérapeutique.
